# Shedding light on the parasite communities and diet of the deep‐sea shark *Deania profundorum* (Smith & Radcliffe, 1912) (Squaliform: Centrophoridae) from the Avilés Canyon (southern Bay of Biscay)

**DOI:** 10.1111/jfb.70292

**Published:** 2026-01-21

**Authors:** Wolf Isbert, Cristina Rodríguez‐Cabello, Francisco E. Montero, Maite Carrassón, Inmaculada Frutos, Ana Pérez‐del‐Olmo

**Affiliations:** ^1^ Instituto Español de Oceanografía (IEO) Santander Spain; ^2^ Unidad de Zoología Marina, Instituto Cavanilles de Biodiversidad y Biología Evolutiva Universitat de València Valencia Spain; ^3^ Alfred Wegener Institute Helmholtz Centre for Polar and Marine Research (AWI) Bremerhaven Germany; ^4^ Departament de Biologia Animal, Biologia Vegetal i Ecologia Universitat Autònoma de Barcelona Barcelona Spain; ^5^ Department of Invertebrate Zoology and Hydrobiology University of Lodz Łódź Poland

**Keywords:** arrowhead dogfish, Bay of Biscay, Northeast Atlantic, parasite infracommunities, prey

## Abstract

Deep‐sea elasmobranchs are less resilient to the increasing scale of anthropogenic impacts such as fisheries, owing to their life‐history traits. The necessity for proper management measures is hampered by the scant knowledge on these taxa and their biology. Here we provide the first comprehensive insight into the parasite infracommunities and trophic habits of the deep‐sea arrowhead dogfish, *Deania profundorum* (Smith & Radcliffe, 1912) from the Avilés Canyon System in the southern Bay of Biscay (Northeast Atlantic). The study revealed rich parasite infracommunities dominated by cestode species represented by both larval and adult stages, as well as larval nematodes, indicating a rather intermediate position of this shark species in the local food web. Sampling years and sexes did not reveal significant differences in host size. Overall prevalence was 89.7% [confidence interval (CI): 72.6–97.8], with an infracommunity mean abundance of 42.2 ± 71.6 (range: 0–292). The infracommunity parameters mean abundance, richness and diversity were associated with host size. Differences in dominance in parasite communities were explained by the factors, year and sex. The composition and structure of parasite communities revealed differences between sampling years, with a significant effect of host size on interannual community similarity. The abundance of the three key discriminating taxa, adult *Deanicola* sp., larval Lacistorhynchidae gen. sp. and larval *Anisakis* sp. (Type I sensu Berland, 1961), increased with host size and revealed a higher parasite burden in larger specimens. Over one third of the sampled specimens revealed empty stomachs. The arrowhead dogfish feeds mainly on bentho‐ and bathypelagic fishes, crustaceans and cephalopods, but the observed prey composition showed no association with either sampling years, sex, or host size. This study underscores the importance of providing new data to the limited existing knowledge, which is necessary for the potential use of parasites as biological indicators. Such information can help infer long‐term feeding niches and elucidate ecological roles within local food webs of hard‐to‐access species, such as deep‐sea sharks.

## 1 INTRODUCTION

The absence of sharks can affect local ecosystems through trophic cascades and mesopredator release, potentially leading to trophic downgrading, highlighting their importance for ecosystem functioning and the re‐establishment of ecosystem resilience (Dedman et al., 2024; Heupel et al., 2014). However, the functional understanding of their ecological roles within marine communities is often limited, specifically in difficult‐to‐study environments such as the deep sea (Dedman et al., 2024). The dearth of relevant information on elasmobranchs impedes the development of proper management and conservation measures (Gallagher et al., 2012; Santos et al., 2020).

**FIGURE 1:**
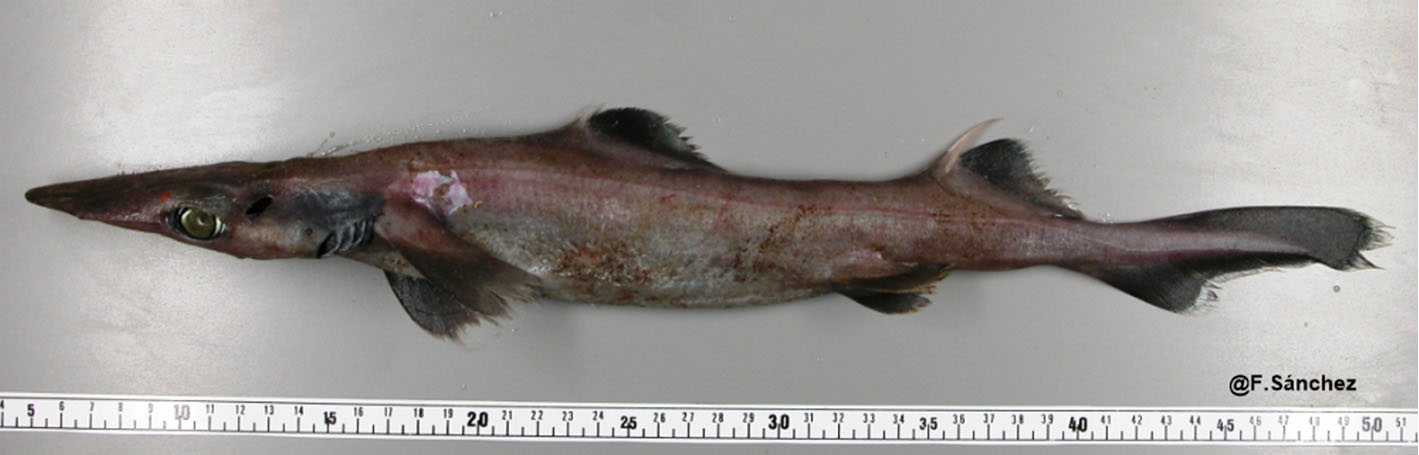
Specimen of *Deania profundorum* caught in the southern Bay of Biscay, still partly frozen shortly before dissection.

Species of the Centrophoridae (Squaliformes) are distributed from the tropics to warm temperate waters on continental and insular shelves and slopes, from upper to middle bathyal depths across the Atlantic, Indian and Pacific oceans (Musick et al., 2004; Nelson, 2006). The genus *Deania* Jordan & Snyder, 1902 is represented by four species, *Deania calceus* (Lowe, 1839), *Deania hystricosa* (Garman, 1906), *Deania profundorum* (Smith and Radcliffe, 1912) and *Deania quadrispinosa* (McCulloch, 1915), and is distributed in all oceans (Froese & Pauly, 2025; Musick et al., 2004). However, recently two studies have indicated that *D. calceus* and *D. hystricosa* are synonyms (Marrero et al., 2023; Rodríguez‐Cabello et al., 2020). The arrowhead dogfish, *D. profundorum* (Figure 1), has been recorded from the West Pacific, the Indian and both sides of the North and South Atlantic (Compagno, 1998; Froese & Pauly, 2025; Nelson, 2006), the Bay of Biscay (Cantabrian Sea) being the northernmost extension in the Northeast Atlantic (Sanjuán et al., 2012). This species usually occurs in habitats on or near the seabed at depths between 270 and 1800 m (Compagno, 1984).

Owing to their life‐history traits (e.g., slow growth rates, late maturation, low fecundity and long gestation periods), deep‐water shark populations can be highly impacted by fishing activities (Dunn et al., 2010; Simpfendorfer & Kyne, 2009). As centrophorids are occasionally represented in considerable numbers as by‐catch in demersal fisheries (Fauconnet et al., 2019), the global population of *D. profundorum* is estimated to have declined by up to 29% over the past three generations (Finucci et al., 2020).

Apart from potential anthropogenic impacts on *Deania* spp. (e.g., Lozano‐Bilbao et al., 2018; Rodríguez‐Cabello & Sánchez, 2017; Santos et al., 2020), only few biological and ecological aspects of *D. profundorum* have been published to date, including studies on reproduction (Sousa et al., 2009), diet (Carvalho Ramos, 2018; Compagno, 1984; Ebert et al., 1992) or taxonomical issues (Rodríguez‐Cabello et al., 2020; Sanjuán et al., 2012; Stefanni et al., 2021). Scant available data, including samples from the Northeast Atlantic, refer to teleosts, cephalopods and crustaceans as main prey of the benthopelagic centrophorids such as *D. profundorum* (Carvalho Ramos, 2018; Compagno, 1984; Ebert et al., 1992). Knowledge on the diet and feeding habits remains scarce, especially for deep‐water species, often due to low sample sizes and empty stomachs (Bowman, 1986; Dunn et al., 2010; Martin & Mallefet, 2023; Wetherbee & Cortés, 2004).

Parasites as bioindicators have been applied successfully in many studies concerning fisheries science (Timi, 2025) and also with regard to the parasite‐prey relationship in marine hosts (e.g., Lafferty, 2013; Rasmussen & Randhawa, 2018; Silva et al., 2017). As indicated by MacKenzie and Abaunza (1998), directly and trophically transmitted parasites can be used as bioindicators as hosts are only infected by parasite species in geographical areas suitable for this parasite transmission (availability of all hosts relevant for its life cycle). If hosts are found outside these endemic areas but are infected with this parasite, it can be concluded that hosts have previously remained within the endemic area of the parasite (MacKenzie & Abaunza, 1998). In addition to potential host movement patterns, the analysis of trophically transmitted parasites, which accumulate over a host lifetime, can reveal information on long‐term feeding habits, host's trophic position in the food web and prey composition consumed by host species (Lafferty et al., 2008). Therefore, this parasite community analysis complements the information on feeding habits of hosts, as stomach and intestine contents of a host represent only a short temporal period of feeding activity, and often their content is lost through the digestion process and partially during sampling with fishing gear (regurgitation) (Lafferty et al., 2008). Due to their close association with the host's feeding habits, knowledge on the life‐cycle pathways and transmission patterns of parasites also provides information on food web structure (Cirtwill et al., 2016; Dallarés et al., 2014; Knudsen et al., 2010; Lafferty et al., 2008; MacKenzie, 2002; Marcogliese, 2002; Muñoz et al., 2002). Additionally, directly transmitted parasites such as monogeneans not only indicate migratory behaviour but also density patterns of their host populations (Grutter, 1998; Sasal, 2003). The use of parasites as biological indicators has been particularly recommended for deep‐sea and rare host species (Caira, 1990; MacKenzie & Abaunza, 1998), and the basic information required on the relationships between parasites and prey composition has already been obtained for several deep‐water species (e.g., Constenla et al., 2015; Dallarés, Carrassón, & Schaeffner, 2017; Dallarés, Pérez‐del‐Olmo, et al., 2017; Isbert et al., 2015; Klimpel, Palm, & Seehagen, 2003; Klimpel, Seehagen, & Palm, 2003; Pérez‐i‐García et al., 2017).

Available information on parasites in species of the genus *Deania* derives from the Pacific, Northeast and Central East Atlantic, comprising cestodes, nematodes and a few monogeneans (Klimpel et al., 2009; Palm & Schröder, 2001; Schröder, 1999). Even though based on few host specimens, data for *D. profundorum* from the Central East Atlantic revealed a rich parasite fauna (Caira & Pickering, 2013; Palm & Schröder, 2001; Schröder, 1999) and highlighted its potential as an excellent indicator of host biology.

The supposed important role of elasmobranchs in deep‐water communities of the southern Bay of Biscay (Sánchez et al., 2008) requires further insights. The logistical constraints and expenses associated with research expeditions carried out at great ocean depths often complicate the collection of deep‐water organisms, and only small sample sizes are often available (Cailliet et al., 2001; Klimpel et al., 2009). The present study on *D. profundorum* provides pioneering results for this species in the Bay of Biscay by contributing new data to the scant information available for the genus, not only on parasite communities and the recent feeding habits (stomach contents) but also on their combined use to infer the long‐term feeding niche of this shark species and to elucidate its position in the local food web. These data on the parasite communities, diet composition and their potential relationship represent a further step towards improved knowledge of the biology and ecology of this genus/species and the functioning of local deep‐sea ecosystems in the future.

## 2 MATERIALS AND METHODS

### 2.1 Study area

**FIGURE 2:**
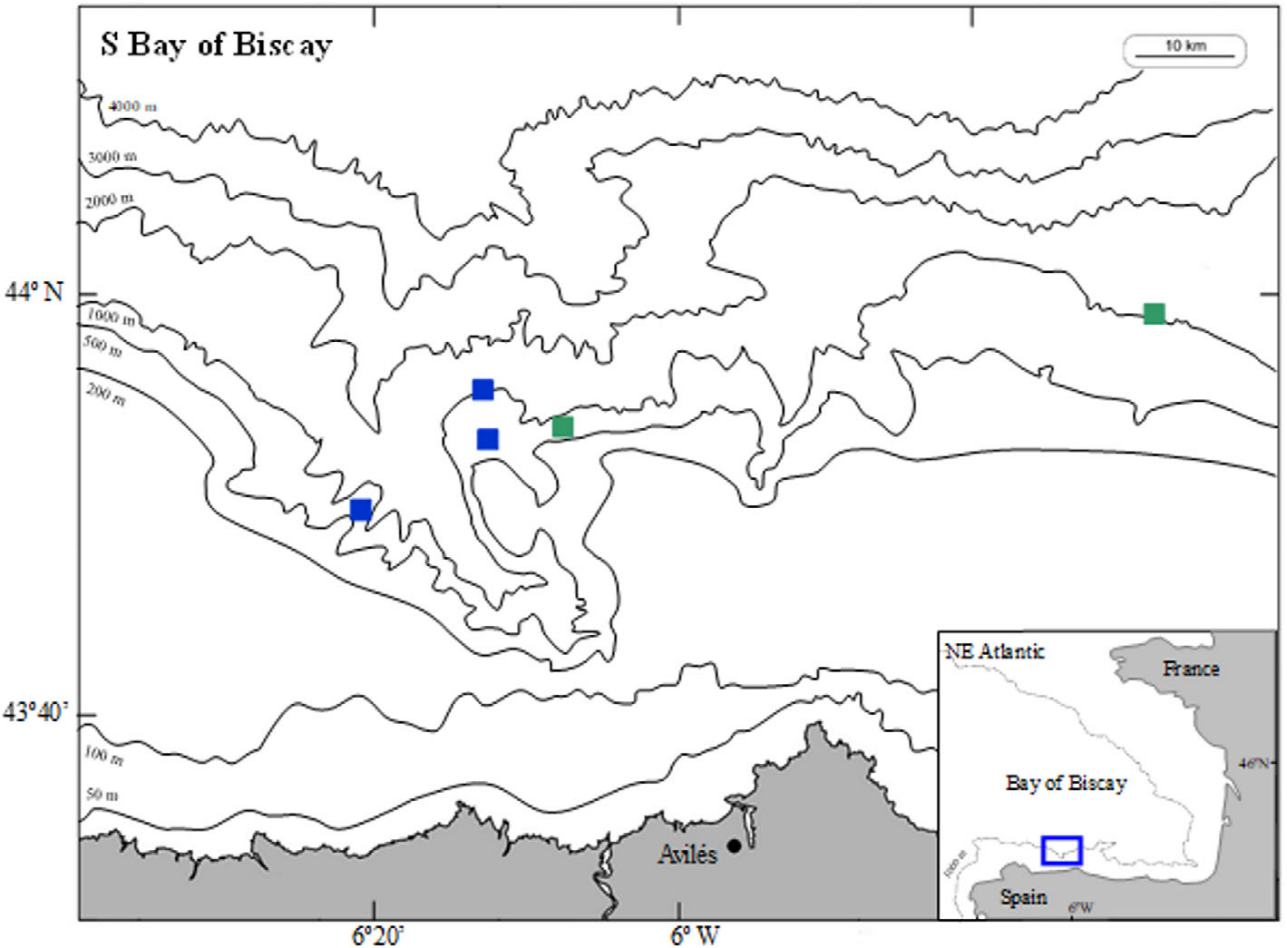
Sampling stations of the arrowhead dogfish, *Deania profundorum*, in the Avilés Canyon System, southern Bay of Biscay (Northeast Atlantic) during INDEMARES cruises in 2010 (blue squares) and 2011 (green squares).

This work has been developed in the Avilés Canyon System (southern Bay of Biscay, Northeast Atlantic), located close to the northern Spanish coast (ca. 7 miles, Figure 2), intersecting the Cantabrian Sea continental shelf at ca. 140 m to ca. 4700 m depth. This area is characterized by continental input of sediments and organic matter by freshwater runoff. Furthermore, poleward currents in winter and equatorward currents in spring/summer produce highly productive upwelling events and phytoplankton blooms (González‐Quirós et al., 2003; Louzao et al., 2010; Ruiz‐Villarreal et al., 2004), contributing to the regional abundance in food resources and enhancing the high species richness of the Avilés Canyon System, including biodiversity hotspots with sponge aggregations and coral reefs in certain regions (Ríos et al., 2022). The canyon is nowadays included in the Natura 2000 network, and recorded diverse communities are considered to be distinctly different from those of the surrounding continental shelf (Louzao et al., 2010).

### 2.2 Sampling

Within the framework of INDEMARES 0710 and INDEMARES 0511 surveys on board, R.V.s *Thalassa* and *Vizconde de Eza*, respectively, specimens of *D. profundorum* were sampled by means of a bottom trawl net (GOC‐73, mesh‐size 10 mm, haul duration 30 min) at five haul stations in July 2010 and May 2011 (Table 1). Specimens were identified on board and immediately frozen at −25°C for further inspection. In the laboratory, prior examination and dissection, each specimen was defrosted, sexed and total body length (TL) was recorded to the nearest 0.1 cm.

**TABLE 1:** Haul characteristics for individuals of *Deania profundorum* caught in two oceanic surveys.

Cruise	Haul	Date	Position^a^		Depth^b^	*n* (♂/♀)	TL (cm)	
		d/m/y	N	W	(m)		Range	Mean ± SD
INDEMARES 07/10	4	22/07/2010	43°55.24’	06°13.79’	982–1007	1 (0/1)	–	54.0 ± 0.0
	5	23/07/2010	43°49.82’	06°21.08’	667–1258	5 (5/0)	29.7–61.0	48.7 ± 12.5
	8	26/07/2010	43°53.01’	06°13.20’	608–855	7 (3/4)	27.9–49.5	36.0 ± 8.5
						**13 (8/5)**	**27.9–61.0**	**42.3 ± 11.8**
INDEMARES 05/11	5	15/05/2011	43°53.29’	06°07.77’	578–624	10 (5/5)	30.0–70.4	43.4 ± 11.2
	7	19/05/2011	43°58.80’	05°28.76’	990–1032	6 (2/4)	26.5–50.3	36.6 ± 9.7
						**16 (7/9)**	**26.5–70.4**	**40.9 ± 10.9**
					**Total**	**29**	**26.5–70.4**	**41.5 ± 11.1**

*Note*: Number of *D. profundorum* individuals, in total and by sex, and total body length (TL) range and mean value for each haul.

Abbreviations: n, number of individuals; SD, standard deviation.

^a^ Haul start point.

^b^ Depth at haul start and end point.

### 2.3 Parasitological examination

In the laboratory, the analysis of both the whole body and its parts (external and internal) was performed. Furthermore, each organ and the entire musculature were inspected separately for parasites under a stereomicroscope. All metazoan parasites were collected and preserved in 70% ethanol or fixed in 4% borax‐buffered formalin for subsequent identification. Monogeneans and cestodes were stained with iron acetocarmine, dehydrated in alcohol series, cleared in dimethyl phthalate and mounted on slides in Canada Balsam (Georgiev et al., 1986). Nematodes were examined in 100% glycerine after dehydration in graded glycerine ethanol series. All parasites were identified to the lowest taxonomic level possible using specialized identification keys (e.g., Anderson et al., 2009; Khalil et al., 1994; Moravec, 1994; Palm, 2004; Yamaguti, 1963) and subsequently counted.

Ecological terminology for the quantitative description of the parasite populations and communities follows Bush et al. (1997). Prevalence of single parasite species per host, provided in percentage (% P) with Sterne's exact confidence interval (CI) (< 1000 samples; CI; 95%), is the number of hosts infected with a particular parasite taxon divided by the number of hosts examined; mean abundance (MA) is the total number of parasite individuals of a particular parasite taxon divided by the number of hosts examined in the sample. Parasite infrapopulation represents all individuals of a parasite taxon in an individual host, whereas the infracommunity comprises all infrapopulations in a single host (Bush et al., 1997; Timi, 2025). Five infracommunity parameters were calculated: (i) infracommunity richness, representing the number of parasite species present per host; (ii) infracommunity abundance (also referred to as total abundance), defined as the total number of parasite individuals of all species per host; (iii) overall prevalence considering all parasite species, calculated as the proportion of hosts infected by at least one parasite individual of any parasite species divided by the total number of hosts examined; (iv) Brillouin diversity index and (v) the Berger‐Parker dominance index (both in Lincoln et al., 1998), both estimated per host.

### 2.4 Diet analysis

All stomachs were weighed (wet weight), dissected and their prey items were identified to the lowest possible taxonomic level using a stereomicroscope. Identification keys were used for fish (Lloris, 2015; Whitehead et al., 1986), and otoliths found in the stomach contents were identified using the AFORO web (http://www.cmima.csic.es/aforo/; Lombarte et al., 2006). Crustaceans and cephalopods were identified following Crosnier and Forest (1973), Baker et al. (1990), Guerra (1992), Keable (2006) and Jereb and Roper (2010). The contribution of each food item to the diet was expressed as frequency of occurrence (% F), percentage by number (% N) and percentage by mass (% W) following Hyslop (1980). The frequency of occurrence was determined as the number of stomachs with prey item ‘i’ divided by the number of all full stomachs (stomachs with food and prey items). The (% N) and (% W) were calculated as the number or mass of prey item ‘i’ divided by the total number or mass of all prey items, expressed as a percentage. The index of relative importance per item I_RI_ (Pinkas et al., 1971) was calculated to facilitate comparisons between samples of other studies. This index is expressed as the sum of I_RI_ indices in all prey: I_RI_ = (I_RIi_ ƩI^−1^_RIi_).

### 2.5 Data analysis

Parasite infrapopulations and infracommunities (i.e., parasites population or community in an individual fish) were used as replicate samples for the analyses. Parasite taxa with % P < 5 were excluded from the analyses to focus on more common taxa and to reduce the background noise. For the quantitative diet comparison, the number of prey items per taxon in each individual fish was used. For the analyses, individual prey items were summed into higher‐level taxonomic groups due to the overall low numerical representation of single prey taxa.

Data on host TL were tested for normality and variance of homogeneity [Kolgomorov‐Smirnov test (KS test), Brown‐Forsythe test (BF test)] followed by parametric test (*t*guion “‐” test) to assess potential effects by the factor year or sex. The relationship between sex and host TL was considered because females of some elasmobranch species are known to grow larger than males, and both sex and TL are potential drivers of variation in prey quantity and composition. The host sex ratio between years was assessed using a Pearson χ^2^. Potential differences in the overall prevalence and infracommunity abundance, as well as in the prevalence and abundance of parasite taxa between years or sexes, were analysed using Fisher's exact test and Bootstrap‐2 sample test, considering the skewed data distribution, typical of parasite communities. Potential effects of the factor year and sex, each with TL as covariate, were tested on parasite infracommunity parameters using generalized linear models (GLM, negative binomial), for infracommunity richness and abundance, and general linear models (GLM) for Brillouin diversity and Berger‐Parker dominance indices, as the latter are continuous response variables. Therefore, values of the Brillouin diversity and Berger‐Parker dominance indices were ln (*x* + 1) transformed and tested for normality and variance of homogeneity (KS test, BF test) before being used in GLMs. The potential effects of factor year and sex on parasite taxa and on higher‐level taxonomic groups of prey items (total prey item number, crustaceans, cephalopods, fish) were also tested using negative binomial GLMs, with TL as covariate. The Spearman's correlation coefficient (r_s_ or rho) is used as a statistical measure to assess the strength and direction of the monotonic relationship between two variables. In the present case, this coefficient was used to assess the relationship between TL and infracommunity parameters, abundance of single parasite taxa and higher‐level prey item groups. The closer r_s_ is to +/−1, the stronger the relationship between the data. All these analyses were carried out using PASW Statistics 18.0.0 (https://www.ibm.com/products/spss-statistics) and Quantitative Parasitology (QP 3.0; Rozsa et al., 2000).

Similarity in parasite community and prey item assemblages between sexes and years was analysed using PRIMER version 6 software (Anderson et al., 2008). Abundance data of parasite community and prey items were transformed after *Hellinger* using the R package (vegan; RStudio 2024.04.0 Build 735). A dummy species consisting of a single parasite individual or prey item was added to all shark specimens to account for the absence of non‐parasitized individuals or empty stomachs. These data were used as the basis for permutational multivariate analyses of covariance (PERMANCOVA; Anderson, 2001), with TL (log transformed) as covariate and year and host sex as fixed factors to assess their effects on parasite community and prey assemblage composition and structure while accounting for host size. Permutation *p*‐values were obtained under a reduced model of permutation of raw data 9999 permutations and the SUM OF SQUARES TYPE I (sequential), based on the Bray‐Curtis resemblance matrix. A permutational analysis of multivariate dispersions (PERMDISP) was conducted assessing homogeneity among infracommunities and prey assemblages between sampling years and sexes. The distances were calculated to centroids and *p*‐values for the F‐ratio obtained using permutations. When PERMANCOVA detected significant differences attributable to ‘year’ or ‘host sex’, a SIMPER analysis was used to identify key discriminating taxa on the basis of the overall percentage contribution of each parasite taxon to the average similarity within and dissimilarity between sampling years or sexes.

## 3 RESULTS

### 3.1 Host

Considering the total number of analysed shark specimens (29), the mean value for TL was 41.5 ± 11.1 cm and the sex ratio was 1:0.9 in favour of males [(♂: 15/♀:14), see Table 1]. The TL of processed individuals complied with normality and variance homogeneity requirements [K‐S: d = 0.12, *p* > 0.2; Brown‐Forsythe (sex): *F* = 1.20, *p* = 0.28; Brown‐Forsythe (year): *F*: 0.95, *p* = 0.33].  Neither TL nor sex ratios differ significantly (*t* = 0.33, *p* = 0.74; Pearson χ^2^ = 0.91, *p* = 0.34, df = 1) between both years of sampling [July 2010 (*n* = 13), May 2011 (*n* = 16)]. Additionally, TL did not differ significantly between both sexes (*t* = 1.32, *p* = 0.19).

### 3.2 Parasite communities

In the 29 analysed specimens of *D. profundorum*, a total of 1224 parasite individuals belonging to nine different taxa were found (Table 2). Overall prevalence was 89.7%, with an infracommunity mean abundance (i.e., total mean abundance) of 42.2 ± 71.6 (range 0–292) parasite individuals (Table 3).

**TABLE 2:** Site in host (i.e., microhabitat within the host), prevalence [% P (95% confidence interval)], mean abundance (MA ± standard deviation)] of parasite species recovered from *Deania profundorum* in the Avilés Canyon (southern Bay of Biscay).

Parasite	Site in host	P %	MA
Monogenea			
*Squalonchocotyle spinacis*	gi	3.4 (0.0–17.7)	0.03 ± 0.2
*Squalotrema* sp.	nc	13.8 (3.8–31.7)	0.2 ± 0.5
Cestoda			
*Aporhynchus* cf. *menezesi*	si	10.3 (2.2–27.4)	0.1 ± 0.3
*Deanicola* sp.	st, si	75.9 (56.5–89.7)	4.4 ± 6.0
Lacistorhynchidae gen. sp.^a^, ^b^	st, si, m, go	79.3 (60.3–92.0)	4.3 ± 5.0
*Sphyriocephalus* sp.^a^	st	6.9 (0.8–22.7)	0.1 ± 0.3
Tetraphyllidea fam. gen sp.^a^, ^b^	l, si	13.8 (3.9–31.7)	0.4 ± 1.4
Nematoda			
*Anisakis* sp. (Type I sensu Berland, 1961)^a^, ^b^	l, sp., k, go, st, si, m	79.3 (60.3–92.0)	32.5 ± 69.6
*Hysterothylacium* sp.^a^, ^c^	st, si	10.3 (2.2–27.4)	0.1 ± 0.3

Abbreviations: gi, gills; go, gonads; k, kidney; l, liver; m, muscle; nc, nasal cavity; si, spiral intestine; sp, spleen; st, stomach.

^a^ Larval stages.

^b^ Encapsulated larvae.

^c^ Lumen.

**TABLE 3:** Comparative data for parasite infracommunity parameters in total, and by sampling years (2010, 2011) and sex, for *Deania profundorum* from the Avilés Canyon (southern Bay of Biscay).

	Total	2010	2011	Male	Female
	Mean ± SD	Mean ± SD		Mean ± SD	
Infracommunity richness	2.9 ± 1.5	2.6 ± 1.8	3.1 ± 1.1	2.7 ± 1.8	3.1 ± 1.0
Infracommunity abundance	42.2 ± 71.6	32.2 ± 60.8	50.3 ± 80.4	70.0 ± 92.2	27.4 ± 49.8
Brillouin diversity index	0.6 ± 0.3	0.5 ± 0.4	0.5 ± 0.3	0.5 ± 0.4	0.6 ± 0.3
Berger‐Parker dominance index	0.6 ± 0.3	0.5 ± 0.3	0.7 ± 0.2	0.5 ± 0.3	0.6 ± 0.2
Overall prevalence	89.7 (72.6–97.8)	76.9 (48.0–93.4)	100.0 (76.8–100.0)	80.0 (53.4–94.3)	100.0 (76.8–100.0)

*Note*: Community parameters (mean ± standard deviation); overall prevalence [% P (95% confidence interval)].

Five of the nine detected taxa were represented by larval stages, which comprised the majority of all identified parasite individuals (88.7%) (Table 2). Nematodes and monogeneans were represented by larval and adult stages, respectively, whereas cestodes showed a balanced ratio between larval and adult stages. Six out of nine taxa are recorded for the first time in *D*. *profundorum*: cestodes *Aporhynchus* cf. *menezesi* Noever, Caira, Kuchta & Desjardins, 2010 (Trypanorhyncha, Aporhynchidae), *Sphyriocephalus* sp. (Trypanorhyncha, Sphyriocephalidae), Tetraphyllidea fam. gen sp., monogeneans, *Squalonchocotyle spinacis* (Goto, 1894) Cerfontaine, 1899 and *Squalotrema* sp. (Monopisthocotylea, Monocotylidea), and nematodes of the genus *Hysterothylacium* (Ascaridoidea, Raphidascaridae). *S. spinacis* was the only species with a P% <5% and was therefore the only excluded one from further analyses.

Overall prevalence and infracommunity abundance per year did not differ significantly (Fisher's test: *p* = 0.08; Bootstrap‐2 sample: *t* = −0.45, *p* = 0.65; Table 3). Among all parasite taxa analysed, only Lacistorhynchidae showed a significantly higher mean abundance in 2011 compared to 2010 (Fisher's test: *p* = 0.36; Bootstrap‐2 sample: *t* = −2.91, *p* = 0.01; the remaining taxa, Fisher's test: *p* = 0.19–1.0; Bootstrap‐2 sample: *t* = −0.45–2.91, *p* = 0.28–0.65). The GLM concerning the dominance (Berger‐Parker dominance index) revealed a significant difference with respect to sampling years, TL and the interaction between the factor and covariate (Table 4). Overall, the negative binomial GLMs, in which the effect of specimen size was partialled out, revealed that host TL explained differences in infracommunity abundance as well as in the abundance of the most prevalent and abundant parasite taxa (i.e., *Anisakis* sp. Type I sensu Berland, 1961, Lacistorhynchidae gen. sp. and *Deanicola sp*.) (Table 4). In the case of *Deanicola* sp., the differences in abundance were explained by the sampling year and host TL (Table 4).

**TABLE 4:** Results obtained from generalized [negative binomial GLM (neg. binom.)] and general linear models (GLM) on parasite infracommunity parameters and on most prevalent and abundant parasite taxa detected in *Deania profundorum* from the Avilés Canyon System (southern Bay of Biscay).

GLM (neg. binom.)	χ^2^	df	*p*‐Value	GLM (neg. binom.)	χ^2^	df	*p*‐Value
Infracommunity abundance							
Year	2.200	1,25	0.138	Sex	0.063	1,25	0.801
TL	49.539	1,25	<0.001	TL	42.995	1,25	<0.001
Year × TL	0.651	1,25	0.421	Sex × TL	0.005	1,25	0.944
Infracommunity richness							
Year	0.964	1,25	0.326	Sex	0.778	1,25	0.378
TL	2.541	1,25	0.111	TL	2.025	1,25	0.155
Year × TL	0.688	1,25	0.407	Sex × TL	0.457	1,25	0.499

GLM	*F*	df	*p*‐Value	GLM (neg. binom.)	*F*	df	*p*‐Value
Brillouin diversity index							
Year	1.923	1,25	0.178	Sex	0.248	1,25	0.623
TL	2.915	1,25	0.100	TL	4.394	1,25	0.046
Year × TL	1.415	1,25	0.245	Sex × TL	0.742	1,25	0.397
Berger‐Parker dominance index							
Year	12.913	1,25	0.001	Sex	11.842	1,25	0.020
TL	12.846	1,25	0.001	TL	5.518	1,25	0.027
Year × TL	9.042	1,25	0.006	Sex × TL	9.039	1,25	0.006

GLM (neg. binom.)	χ^2^	df	*p*‐Value	GLM (neg. binom.)	χ^2^	df	*p*‐Value
*Anisakis* sp.							
Year	0.236	1,25	0.627	Sex	0.114	1,25	0.735
TL	72.582	1,25	<0.001	TL	70.001	1,25	<0.001
Year × TL	0.024	1,25	0.878	Sex × TL	0.620	1,25	0.431
Lacistorhynchidae							
Year	1.148	1,25	0.284	Sex	1.732	1,25	0.188
TL	9.363	1,25	0.002	TL	8.230	1,25	0.004
Year × TL	0.101	1,25	0.751	Sex × TL	1.675	1,25	0.196
*Deanicola* sp.							
Year	5.448	1,25	0.020	Sex	1.115	1,25	0.291
TL	6.271	1,25	0.012	TL	2.509	1,25	0.113
Year × TL	3.807	1,25	0.051	Sex × TL	0.962	1,25	0.327

*Note*: GLM (neg. binom.) and GLM were conducted on two single factors, year and sex, respectively, with total body length (TL) used as covariate.

**FIGURE 3:**
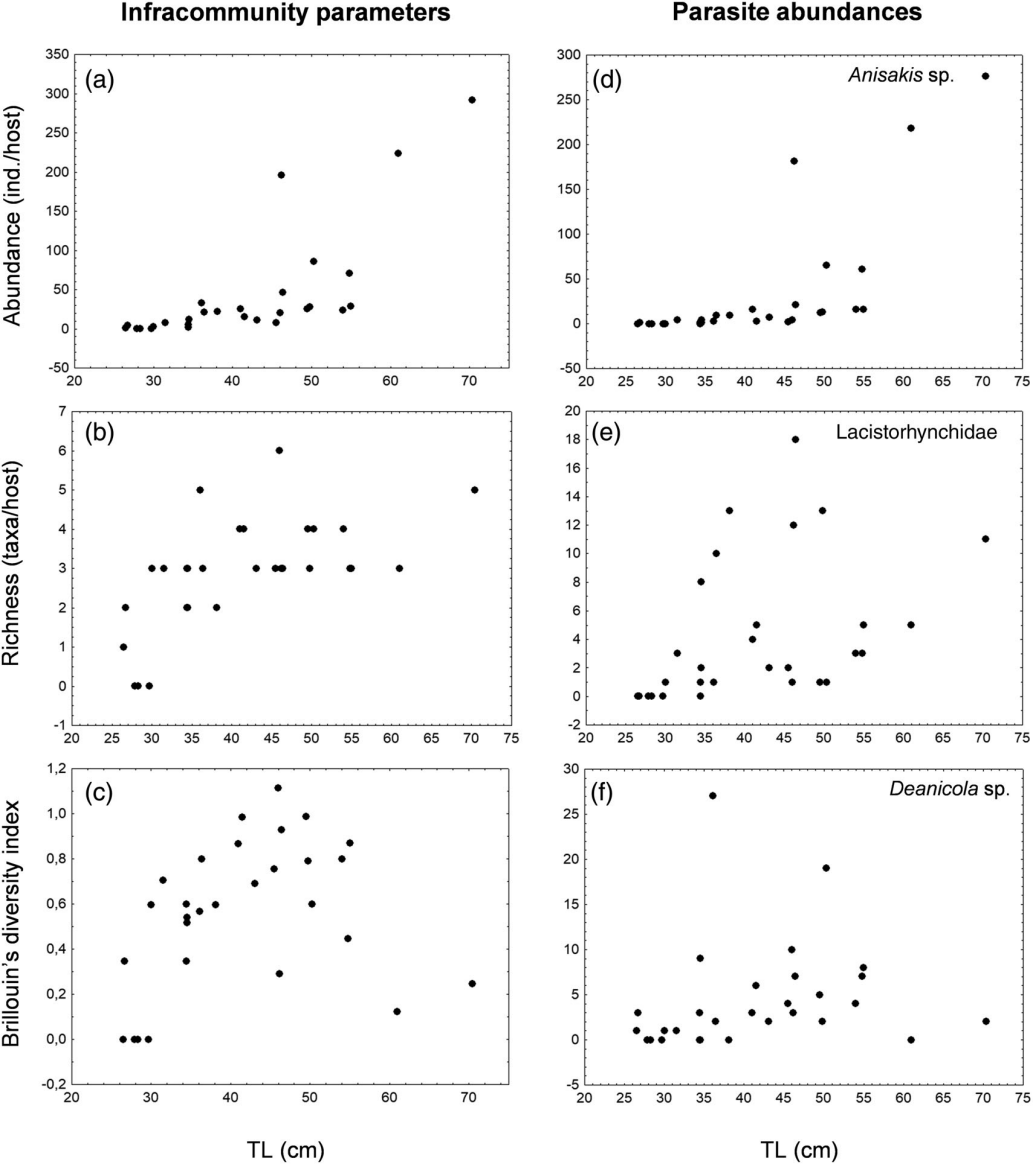
Scatterplots for the relationship of the host body size (TL), with infracommunity parameters and abundances of more relevant parasite taxa found in *Deania profundorum* from the Avilés Canyon system (southern Bay of Biscay). (a) Infracommunity abundance; (b) infracommunity species richness; (c) Brillouin's diversity index; (d–f) abundance of *Anisakis* sp. Type I (sensu Berland, 1961) (d), Lacistorhynchidae gen. sp. (e), *Deanicola* sp. (f).

The shark size was significantly associated with the infracommunity abundance (r_s_ = 0.874, *p* < 0.0001), richness (r_s_ = 0.639, *p* = 0.0002) and Brillouin diversity index (r_s_ = 0.419, *p* = 0.02) (Figure 3), whereas Berger‐Parker dominance index showed no significant relationship with TL (r_s_ = 0.318, *p* = 0.09; not plotted in Figure 3). The abundances of the three most prevalent and abundant parasite taxa (Table 2) were significantly associated with shark size (*Anisakis* sp. Type I sensu Berland, 1961, r_s_ = 0.888, *p* < 0.0001; Lacistorhynchidae gen. sp. r_s_ = 0.622, *p* = 0.0003 and *Deanicola* sp. r_s_ = 0.451, *p* = 0.01) (Figure 3).

Overall prevalence, infracommunity abundance and the abundance of the most prevalent parasite taxa did not show significant differences between sexes (Fisher's test: *p* = 0.22–1.0; Bootstrap‐2 sample: *t* = −1.10–0.943, *p* = 0.29–0.85; Table 3). The negative binomial GLM and the GLM on infracommunity abundance and diversity (i.e., Brillouin diversity index) respectively, in which TL was partialled out, revealed that host TL only explained the observed differences (Table 4). The differences in dominance (i.e., Berger‐Parker dominance index) were explained by both host sexes and host TL, resulting in an interaction between the factor and covariate (Table 4). Host sex did not explain differences in the three most prevalent and abundant parasite taxa, whereas host TL could partly explain differences in *Anisakis* sp. and Lacistorhynchidae.

The PERMANCOVA with TL as a covariate provided strong support for the differentiation of parasite infracommunities composition and structure associated with the sampling year but not with sex (Table 5). Additionally, the analysis showed a significant effect of host TL on community similarity, both when analysing single factors and when considering both factors together, resulting in interaction between the covariate and the factors (Table 5). In contrast, PERMDISP did not detect any heterogeneity of dispersion with respect to either of the factors analysed, year and sex (Table 5). The SIMPER procedure identified the most prevalent and abundant taxa (larval stages of the nematode *Anisakis* sp. and Lacistorhynchidae and adult stages of *Deanicola* sp.) as key discriminating taxa due to their strong contribution to the similarity among infracommunities in 2010 and 2011, respectively (cumulative contribution: 97.2% and 99.4%). The average similarity of infracommunities in 2010 was distinctly lower (35.0%) than in 2011 (58.5%), whereas the overall dissimilarity between parasite communities of both sampled years was 54.9%, with the same three taxa contributing most to the community dissimilarity (82.1%).

**TABLE 5:** Results obtained from PERMANCOVA and PERMDISP on the composition and structure of the parasite community and prey items detected in *Deania profundorum* from the Avilés Canyon System (southern Bay of Biscay).

	PERMANCOVA				PERMDISP		
Parasite infracom.	df	MS	Pseudo‐*F*	*P(perm)*	df1\df2	*F*	*P(perm)*
TL	1	14,939.0	19.268	<0.001	1\27	6.033	0.070
Year	1	3068.3	3.958	0.009			
Year × TL	1	2356.8	3.040	0.029			
Residual	25	775.3					
TL	1	14,939.0	17.033	<0.001	1\27	1.854	0.332
Sex	1	1757.8	2.004	0.114			
Sex × TL	1	1123.4	1.281	0.294			
Residual	25	877.1					
TL	1	14,939.0	22.918	<0.001			
Year	1	2562.5	3.931	0.010			
Sex	1	1757.8	2.697	0.050			
Year × TL	1	1612.5	2.474	0.073			
Sex × TL	1	905.7	1.389	0.249			
Year × sex	1	1403.7	2.153	0.094			
TL × year × sex	1	2877.1	4.414	0.005			
Residual	21	651.83					
**Diet composition**							
TL	1	1573.3	1.344	0.212	1\27	1.752	0.301
Year	1	1292.1	1.104	0.332			
Year × TL	1	449.7	0.384	0.929			
Residual	25	1170.6					
TL	1	1573.3	1.335	0.220	1\27	0.370	0.641
Sex	1	835.3	0.709	0.666			
Sex × TL	1	706.0	0.599	0.773			
Residual	25	1178.6					
TL	1	1573.3	1.247	0.262			
Year	1	1314.7	1.042	0.379			
Sex	1	835.3	0.662	0.707			
Year × TL	1	418.4	0.332	0.941			
Sex × TL	1	757.3	0.600	0.761			
Year × sex	1	510.1	0.404	0.907			
TL × year × sex	1	672.5	0.533	0.812			
Residual	21	1261.8					

*Note:* Analyses were conducted on two single factors, year and sex, as well as on both factors combined, with total body length (TL) used as covariate, and its interaction with the factors considered.

### 3.3 Diet

Of the 29 examined specimens, 37.9% had empty stomachs, but none of the stomachs was regurgitated. The number of detected prey items in the examined specimens with full stomachs was low, with one and two prey items being found in 61.1% and 33.3% of stomachs, respectively, whereas only one individual (5.6%) exhibited three different prey items in its stomach.

Overall, the diet of *D*. *profundorum* consisted of fishes, crustaceans and molluscs, with fishes as the most relevant prey in terms of % F, % N, % W and I_RI_ (Table 6). This prey group was represented by bentho‐ and bathypelagic species, mostly Myctophidae (*Myctophum punctatum* Rafinesque, 1810), but also Gadidae [*Micromesistius poutassou* (Risso, 1827)] and Lotidae [*Molva macrophthalma* (Rafinesque, 1810)]. The crustaceans exhibited the second highest I_RI_ among the detected prey items, being chiefly represented by caridean decapods. Molluscs were slightly less important than crustaceans in the diet, represented solely by cephalopods.

**TABLE 6:** Data on diet composition, including percentage of occurrence frequency (% F), by number (% N) and by mass (% W), as well as the index of relative importance (I_RI_) of detected prey items, in the stomachs of *Deania profundorum* from the Avilés Canyon System (southern Bay of Biscay).

Prey items	% F	% N	% W	I_RI_
Crustacea	33.33	29.63	6.34	1198.89
Euphausiacea				
*Meganyctiphanes norvergica*	5.56	3.7	0.07	20.97
Caridea	27.78	22.22	5.8	778.28
*Pasiphaea multidentata*	5.56	3.7	2.25	33.09
*Pasiphaea sivado*	5.56	3.7	3.1	37.83
*Pasiphaea* sp.	11.11	7.41	0.13	83.72
Caridea unidentified	11.11	7.41	0.31	85.75
Isopoda				
*Natatolana borealis*	5.56	3.7	0.47	23.19
Mollusca				
Cephalopoda unidentified	16.67	11.11	24.42	592.13
Actinopterygii	72.22	55.56	68.99	8995.05
*Micromesistius poutassou*	5.56	3.7	23.75	152.51
*Molva macrophthalma*	5.56	3.7	0.41	22.83
*Lobianchia gemellarii*	11.11	7.41	9.87	191.99
*Myctophum punctatum*	11.11	7.41	9.2	184.56
Stomiidae unidentified	5.56	3.7	20.37	133.76
Myctophiformes unidentified	11.11	7.41	2.76	113
Actinopterygii unidentified	33.33	22.22	2.63	828.31
Others				
Unidentified	5.56	3.7	0.26	22.04

No significant correlation was found between the TL and the number of higher‐level taxonomic groups, that is, fish prey (r_s_ = 0.115, *p* = 0.5503), carideans (r_s_ = −0.2092, *p* = 0.2760), cephalopods (r_s_ = −0.0270, *p* = 0.8891) and total prey item number (r_s_ = −0.1426, *p* = 0.4605). The negative binomial GLMs, performed for total number of prey items and abundance of higher prey taxa groups, in which the effect of shark size was partialled out, did not show significant differences with respect to the year of sampling and host sex, nor any effect of host TL (Table 7). The PERMANCOVA, run with TL as a covariate, did not reveal any significant differences between years or sexes nor any effect of TL on the composition and structure of the detected diet (Table 5). PERMDISP did not detect any heterogeneity of dispersion with respect to both analysed factors, year or sex (Table 5).

**TABLE 7:** Results obtained from generalized linear models [negative binomial GLM (neg. binom.)] on main prey taxa groups detected in the stomachs of *Deania profundorum* from the Avilés Canyon System (southern Bay of Biscay).

GLM (neg. binom.)	χ^2^	df	*p*‐Value	GLM (neg. binom.)	χ^2^	df	*p*‐Value
Sum diet items							
Year	0.242	1	0.623	Sex	0.009	1	0.923
TL	0.672	1	0.412	TL	0.544	1	0.461
Year × TL	0.084	1	0.771	Sex × TL	0.651	1	0.902
Crustacea							
Year	1.492	1	0.222	Sex	0.098	1	0.754
TL	2.029	1	0.154	TL	3.151	1	0.076
Year × TL	1.012	1	0.314	Sex × TL	0.089	1	0.766
Cephalopoda^a^							
Year	x	x	x	Sex	x	x	X
TL	x	x	x	TL	0.003	1	0.955
Year × TL	x	x	x	Sex × TL	x	x	X
Fish							
Year	0.305	1	0.581	Sex	0.196	1	0.658
TL	0.023	1	0.879	TL	0.093	1	0.760
Year × TL	0.239	1	0.625	Sex × TL	0.293	1	0.589

*Note*: Negative binominal generalized linear models (GLM) were conducted on two single factors, year and sex, with total body length (TL) used as covariate, and its interaction with the factors considered.

^a^ Not sufficient data.

## 4 DISCUSSION

To the best of our knowledge, the present study provides the first comprehensive insight into parasite infracommunities and diet data of the deep‐sea shark *D. profundorum*, based on the largest sample size examined to date. Moreover, almost 67% of the parasite taxa are reported for the first time in this shark species, including a potentially new species of monopisthocotylean monogenean.

The infracommunity parameters in *D*. *profundorum* are partly compared with benthopelagic conspecific individuals, congeneric species and species from other selachian genera. For instance, smaller sample sizes of larger individuals from three *Deania* species in the Great Meteor Bank in the Northeast Atlantic (i.e., *D*. *profundorum*, *D. calceus* and *D. hystricosa*) exhibited a wide range of infracommunity mean abundances (between 30.5 and 153.0 parasite individuals; Palm & Schröder, 2001; Schröder, 1999). Specimens of other selachians, such as *Centroscymnus coelolepis* Barbosa du Bocage & de Brito Capello, 1864 (Somniosidae) and *Galeus melastomus* Rafinesque, 1810 (Pentanchidae) from the Balearic Sea (northwestern Mediterranean Sea), exhibited distinctly higher (928.0) or similar infracommunity mean abundances compared to *D*. *profundorum* (40.8), respectively (Dallarés, Padrós, et al., 2017). Although *C. coelolepis* feeds on different and supposedly larger prey (squid, carrion), potentially already highly infected (Iyaji et al., 2009), the prey of *G*. *melastomus* consists mainly of benthic organisms (Dallarés, 2016; Fanelli et al., 2009) compared to the rather benthopelagic prey of *D*. *profundorum*. Generally, it is suggested that fishes with more benthic feeding habits exhibit higher diversity and abundances of parasites compared to fishes with pelagic and benthopelagic feeding habits (Klimpel et al., 2006; Marcogliese, 2002). The benthic lifestyle increases the probability of encountering a wide range of benthic intermediate hosts of larval parasites.

Dallarés (2016) detected infracommunities in *G*. *melastomus* and *C. coelolepis* clearly dominated by single species, exhibiting higher dominance (do) and lower diversity (di) values compared to the present study [*G. melastomus* 0.9 (do), 0.02 (di); *C*. *coelolepis* 0.9 (do), 0.3 (di)]. In the present study, the overall lower dominance and higher diversity values of infracommunities are explained by similar abundances of three taxa: *Anisakis* sp. Type I sensu Berland, 1961, *Deanicola* sp. and Lacistorhynchidae gen. sp. The infracommunity mean richness (Table 3) is slightly higher than in other selachians (*G. melastomus* 1.8; *C. coelolepis* 2.5; Dallarés, 2016), partly from the same geographical area, that is, *ex Etmopterus spinax* 1.2–1.7 (Isbert et al., 2015) and 2.5 (Isbert et al., 2015 estimated from Klimpel, Palm, & Seehagen, 2003), and even higher compared to rather shallow water sharks (< 200 m), such as *Scyliorhinus canicula* and *Squalus acanthias* (0.6–1.7 and 1.6, respectively; Isbert et al., 2015, estimated from Moore, 2001; Henderson et al., 2002), and *S. canicula* (1.17–1.44, Dallarés, Pérez‐del‐Olmo, et al., 2017). Higher infracommunity richness in *Deania* spp. in the present and other studies (3–5; Schröder, 1999; values estimated from published data) could partly reflect the diverse predatory and opportunistic feeding habits assigned to this genus (Ebert et al., 1992; Preciado et al., 2009) and also to geographical differences in prey diversity acting as intermediate hosts (Cirtwill et al., 2016).

Additionally, underwater features are considered as hotspots for aggregations of higher‐level consumers due to enhanced biological production caused by favourable hydrographic conditions (Clark et al., 2010; Morato et al., 2008; Vetter et al., 2010). Consequently, a supposedly enhanced presence of intermediate hosts could result in a higher parasite richness in *Deania* spp. from the Avilés Canyon System (present study) and the Great Meteor Bank (Palm & Schröder, 2001), also suggested for *G. melastomus* from a canyon in the Balearic Sea (northwestern Mediterranean Sea) (Dallarés, 2016). Nevertheless, no conclusion can be drawn, as it lacks knowledge on the parasite community of the arrowhead dogfish from other localities without the influence of underwater features.

The total parasite richness recorded in *D. profundorum* is comparable to other benthic and benthopelagic shark species from deeper and coastal shallow waters (Table 8). Community composition of those benthic and benthopelagic sharks confirmed that cestodes are the most diverse metazoan parasites found in elasmobranchs (Caira & Healy, 2004). In contrast, ectoparasite communities, regarding especially copepods and monogeneans, seem to be richer in rather shallow water sharks (*S. canicula*, *Mustelus manazo*, *S. acanthias*) than in deep‐water sharks (Table 8). However, the latter might also be explained by larger sample sizes in studies on shallow water sharks. Evidence from studies on *Etmopterus granulosus* and *G. melastomus*, where more than 100 specimens were analysed, supports the enhanced presence of monoxenous species such as copepods. In the present study, the recorded presence of two species of monogeneans and the lack of copepods do not reflect the general assertion of the most diverse ectoparasites found in elasmobranchs (Caira et al., 2012). The low infection levels observed are consistent with the idea of some authors that monogeneans are typically scarce in deeper waters (>1000 m) related to the lower density of their host species, a high host specificity of monogeneans and an ineffective larval dispersal (Campbell et al., 1980; de Buron & Morand, 2004; Dykman et al., 2023). In contrast, other studies indicate the presence of monogeneans even in deeper waters (Ñacari et al., 2024), as well as monoxenous taxa such as copepods and cirripedes at depths clearly below 4000 m (de Buron & Morand, 2004). Nevertheless, it should be considered that fewer species and lower abundances of ectoparasites can also be related to the dislodgement during the sampling procedure as they are fixed between scales, skin or gills (e.g., Moore, 2001; Quattrini & Demopoulos, 2016).

**TABLE 8:** Total number of fish examined (*n*), parasite species’ richness and composition of parasite communities in selachians from different habitats and geographic areas.

Host species	*n*	Richness	Community composition	Habitat	Geographical area	Source
*Apristurus nasutus De Buen, 1959*	13	5	2/1/0/2/0/0	Bathydemersal [D]	Off Chile (SE Pacific)	Espínola‐Novelo et al., 2018
*Centroscyllium fabricii (Reinhardt, 1825)*	40	8	3/1/1/3/0/0	Benthopelagic [D]	Canada (NW Atlantic)	Chambers, 2008
*Centroscymnus coelolepis Barbosa du Bocage & de Brito Capello, 1864*	10	8	6/0/0/2/0/0	Benthopelagic [D]	Balearic Sea (W Mediterranean Sea)	Dallarés, 2016
*Galeus melastomus Rafinesque, 1810*	120	15	5/1/1/7/1/0	Benthopelagic [D]	Balearic Sea (W Mediterranean Sea)	Dallarés, Pérez‐del‐Olmo, et al., 2017
*Deania calcea (Lowe, 1839)*	2	3	2/0/0/1/0/0	Benthopelagic [D]	Great Meteor Bank (NE Atlantic)	Palm & Schröder, 2001
*Deania hystricosa (Garman, 1906)*	8	9	4/1/0/3/0/1	Benthopelagic [D]	Great Meteor Bank (NE Atlantic)	Palm & Schröder, 2001
*Deania profundorum (Smith & Radcliffe, 1912)*	2	7	5/0/0/2/0/0	Benthopelagic [D]	Great Meteor Bank (NE Atlantic)	Palm & Schröder, 2001
*Deania profundorum*	29	9	5/2/0/2/0/0	Benthopelagic [D]	Avilés Canyon System (NE Atlantic)	present study
*Etmopterus granulosus (Günther, 1880)*	120	14	4/5/1/2/2/2	Benthopelagic [D]	Off Chile (SE Pacific)	Espínola‐Novelo et al., 2018
*Etmopterus spinax (Linneaus, 1758)*	37	7	3/2/0/2/0/0	Benthopelagic [D]	Skagerrak (NE North Sea)	Klimpel et al., 2003
*Etmopterus spinax*	30	9	5/1/0/3/0/0	Benthopelagic [D]	Galicia Bank (NE Atlantic)	Isbert et al., 2015
*Etmopterus spinax*	29	7	5/0/1/1/0/0	Benthopelagic [D]	Avilés Canyon System (NE Atlantic)	Isbert et al., 2015
*Etmopterus spinax*	11	2	2/0/0/0/0/0	Benthopelagic [D]	Balearic Sea (W Mediterranean Sea)	Dallarés, 2016
*Heptranchias perlo (Bonnaterre, 1788)*	10	6	3/1/1/1/0/0	Benthopelagic [D]	Great Meteor Bank (NE Atlantic)	Palm & Schröder, 2001
*Mustelus manazo (Bleeker, 1855)*	1038	11	8/0/0/1/2/0	Benthopelagic [S]	Off Japan/Taiwan (NW Pacific)	Yamaguchi et al., 2003
*Scyliorhinus canicula (Linneaus, 1758)*	41	5	1/1/0/3/0/0	Benthic [S]	Balearic Sea (W Mediterranean Sea)	Dallarés, Pérez‐del‐Olmo, et al., 2017
*Scyliorhinus canicula (Linneaus, 1758)*	101	10	2/2/1/3/2/0	Benthic [S]	Off British Isles (NE Atlantic)	Moore, 2001
*Squalus acanthias Linnaeus, 1758*	254	10	2/1/0/2/5/0	Benthopelagic [S]	Off Ireland (NE Atlantic)	Henderson et al., 2002

*Note*: The composition of parasite communities is indicated as the number of species per taxa in the following order: Cestoda/Monogenea/Digenea/Nematoda/Copepoda/others (Isopoda; Thecostraca; Piscicolidae). [S], shallow water species; [D], deep‐water species.

*Squalonchocotyle spinacis* has previously been described in *Etmopterus* spp. from different geographic areas (Di Cave et al., 2003; Espínola‐Novelo et al., 2018; Isbert et al., 2015; Klimpel, Palm, & Seehagen, 2003), where prevalence and mean intensity were notably higher only in *E. spinax* specimens from the Galicia Bank (Northeast Atlantic off the western coast of Spain; Isbert et al., 2015) compared to the infection levels observed in the present study. However, in the same study, no specimens of this monogenean were recorded from *E. spinax* in the Avilés Canyon System.

The markedly different morphology compared to that of the only species previously described, *Squalotrema llewellyni* Kearn & Green, 1983, suggests that *Squalotrema* sp. may represent a new species to science. *Squalotrema* sp. exhibits relatively higher prevalence (of almost 14%) in *D. profundorum*. Nasal cavities of sharks and rays are considered as primary site for the attachment and regularly inhabited by monocotylid species (Caira & Healy, 2004; Chisholm & Whittington, 2012; Justine, 2009; Klimpel, Palm, & Seehagen, 2003). Previous studies proved that monogeneans accumulate during host life (Lo et al., 1998; Morand et al., 2002; Pérez‐del‐Olmo et al., 2008), therefore the lack of correlation of TL and the abundance of *Squalotrema* sp. may be related to the low sample size and relatively narrow host size range.

The prey composition did not reveal any significant differences between the years and sex nor any effect of host TL. The prey composition partly agrees with other studies describing *Deania* spp. as opportunistic, active benthopelagic and high‐trophic level predators preying both above the substratum and in the water column (Cortés, 1999; Ebert et al., 1992; Mauchline & Gordon, 1986; Musick et al., 2004; Preciado et al., 2009; Saldanha et al., 1995; Yano, 1991). Stable isotope analysis of arrowhead dogfish specimens in Portuguese waters also revealed predation on crustaceans and vertical migratory teleosts (Graça Aranha et al., 2023). Additionally, the seemingly accidental ingestion of the isopod crustacean *Natatolana borealis* (Liljeborg, 1851) by *D. profundorum* could suggest opportunistic scavenging habits. This isopod species is a benthic micropredator and scavenger, feeding on dead and dying fish (Keable, 2006), inhabiting the southern Biscay slope (Frutos & Sorbe, n.d., 2014; Ríos et al., 2022), which is known to be well represented in the upper‐slope epibenthic community of the Avilés Canyon System (average biomass: 3291.3 g/km^2^; Módica et al., 2022; Serrano et al., 2012). This coincides with higher abundances of *D. profundorum* (average biomass: 13,820.0 kg/km^2^; Módica et al., 2022) in the upper slope compared to the shelf, shelf break or middle slope of that canyon system. Facultative scavenging is a common and important foraging mode noted in deep‐sea sharks (Drazen & Sutton, 2017; Martin & Mallefet, 2023); therefore, this behaviour can occasionally promote the observed incidental interaction between shark and isopod species.

The herein recorded relatively high proportion of empty stomachs partly agrees with other studies on deep‐sea elasmobranchs (Ebert et al., 1992; Preciado et al., 2009; Wetherbee & Cortés, 2004). Bowman (1986) suggested frequent regurgitation of prey items in deep‐water teleosts and elasmobranchs during haul procedures. Regurgitation could not be detected in the analysed specimens, and few indications of regurgitation were also found by Preciado et al. (2009, 2017). Therefore, empty stomachs could suggest intermittent feeding habits observed in elasmobranchs where feeding in short bouts alternates with long periods of low or no feeding activity (Wetherbee & Cortés, 2004; Yano, 1991). This is confirmed by the analysis of RNA/DNA ratios of different deep‐sea sharks where *D. profundorum* is considered one of the predators that feed less frequently (Graça Aranha et al., 2023).

The detected stomach content did not reveal significant temporal differences and could not be directly assigned to the observed differences in the composition and structure of the parasite community. However, in cases where the information about the diet is limited, trophically transmitted heteroxenous parasites can be a useful indicator of past feeding events (Lafferty et al., 2008). Spatial and temporal patchiness of prey distribution can result in variations in dietary composition of fishes and thus can affect infection by endoparasites (Cirtwill et al., 2016). Existing information on the interannual variability of parasites in deep‐sea fishes is still scarce (Palm & Klimpel, 2008). Nevertheless, especially larval parasite stages are considered to be the permanent evidence of past feeding events and can reflect temporal variations and natural variability in the availability of prey resources (infective stages of trophically transmitted parasites) (Dallarés et al., 2014; Marcogliese, 2002; Perdiguero‐Alonso et al., 2008). Environmental conditions influence the occurrence and distribution of parasites, as regional environmental factors affect directly or indirectly (via intermediate hosts) their transmission success (MacKenzie & Abaunza, 1998; Marcogliese et al., 2016). Larval stages of endoparasites can be particularly relevant given that, although some parasites located in the stomachs may be lost due to regurgitation during the sampling process, they are generally less susceptible to loss compared to monoxenous ectoparasites. In the present study, three out of five cestode taxa were represented by larval stages, resulting in slightly more than half of all cestode individuals detected. This aspect, also reflected by the recorded diet composition (bentho‐ and bathypelagic carideans and teleosts), agrees with the assumption made in a previous study that classified this shark species as a mesotrophic level predator (Graça Aranha et al., 2023). The arrowhead dogfish can instead be identified as a mesopredator, being an intermediate host for those cestode larvae and potential prey for larger elasmobranch taxa in this area (Serrano et al., 2011), which act as definitive hosts for those cestode taxa (e.g., Klimpel, Kellermanns, & Palm, 2008; Palm, 1997). For instance, the new host record of *Sphyriocephalus* sp. postlarvae *ex D. profundorum* adds the arrowhead dogfish to previously recorded different benthic and benthopelagic selachii, as well as epi‐ and mesopelagic and deep‐water teleosts, as intermediate or paratenic hosts (e.g., Dallarés, Carrassón, & Schaeffner, 2017; García et al., 2011; Lester et al., 1988). *Sphyriocephalus viridis* (Wagener, 1854) Pintner, 1913 is described from the Northeast Atlantic, and the present findings could indicate the occurrence of potential definitive hosts, for example, *Dalatias licha* (Dalatiidae) (Dallarés, Carrassón, & Schaeffner, 2017). This shark species has been recorded in the study area (Serrano et al., 2011), and *D. profundorum* has already been recorded as part of its diet (Dunn et al., 2010; Matallanas, 1982).

Additionally, the herein recorded tetraphyllidean and trypanorhynch larvae are commonly omnipresent in marine ecosystems, infecting different species which act as intermediate or paratenic hosts. For Sphyriocephalidae, Aporhynchidae and Gilquiniidae, an oceanic life cycle is assumed, with copepods as first, euphausiids, large decapods, and schooling or other fishes as second intermediate hosts, whereas larger fishes can act as paratenic hosts (Palm, 2004). Several potential second intermediate hosts (euphausiids, decapods, schooling fish) were detected as prey items in *D. profundorum* even though with varying degree of importance (Table 6), assigning *D. profundorum* rather to the role of a paratenic host for those larval stages.

In contrast, along with the intermediate hosts described above for trypanorhynchs, life cycles of Lacistorhynchidae gen. sp. (Trypanorhyncha) involve an obligate predatory finfish or shark species as the third intermediate host, in this case *D. profundorum* (Palm, 2004). The recorded infection sites in the viscera, but especially in the musculature, are consistent with findings of *Grillotia meteori* Palm & Schröder, 2001 ex *Deania* spp., *G*. *acanthoscolex* Rees, 1944 ex *G*. *melastomus* (Dallarés, Padrós, et al., 2017; Palm & Schröder, 2001) and *Grillotia* sp. ex *E*. *spinax* (Isbert et al., 2023). The larval lacistorhynchids (78% of detected larvae) formed large blastocysts deep within the muscle tissue of *D. profundorum*, which is structurally more feasible for this larger host than by small fish species (Palm, 2004). Additionally, almost all lacistorhynchid blastocysts (>90%) were detected in the caudal‐fin musculature, confirming similar observations in other deep‐sea sharks (Dallarés, Padrós, et al., 2017; Isbert et al., 2023). As already suggested, the accumulation of larvae in this site during the host lifetime could affect swimming speed and performance (Dallarés, Padrós, et al., 2017; Isbert et al., 2023; Palm et al., 1994). Together with a presumed longevity of cestode species (Hassan et al., 2002), this could facilitate the predation of heavily infested fish, enhancing the chance of transmission to definitive hosts.

The recorded adults of the trypanorhynch *Deanicola* Beveridge, 1990 (Gilquiniidae) agree with previous findings of *Deanicola minor* and *Deanicola protentus* in *Deania* species, including *D*. *profundorum* (Beveridge, 1990; Palm & Schröder, 2001; Schröder, 1999), and records of plerocerci and adults in different taxa of Squaliformes and Carcharhiniformes (Beveridge, 1990; Beveridge & Justine, 2006; Costa et al., 2014; Klimpel et al., 2009). These aspects, as well as comparable infection levels (prevalence 38%–100%) in the Northeast Atlantic (Palm & Schröder, 2001; Schröder, 1999), highlight *D*. *profundorum* as a common definitive host for *Deanicola* sp. in the Avilés Canyon System. Euphausiids are considered as the obligate intermediate host for Gilquiniidae (Palm, 2004), but the supposedly minor importance of euphausiids within the herein recorded prey items may contradict this. Nevertheless, euphausiids are one of the most abundant crustacean groups in shelf suprabenthic communities, with *Nyctiphanes couchii* (Bell, 1853) as the dominant species at the shelf break of the Avilés Canyon (Frutos et al., 2012, 2017). In deeper bathyal zones, the horizontal impingement of euphausiid swarms from the pelagic to the slope environments, combined with wind‐driven and tidal upwelling circulation, promotes their temporary aggregation in specific areas at the canyon head (Frutos et al., 2017; Mauchline & Gordon, 1991). Thus, despite the appearance of the bathyal euphausiid *Meganyctiphanes norvegica* (M. Sars, 1857) in the upper slope of the Avilés Canyon System (Frutos et al., 2012), its irrelevant occurrence in *D. profundorum* diet indicates rather the supposed opportunistic feeding habit, exploiting locally abundant prey and low abundances of euphausiids in the deep environment at that time (Dunn et al., 2013). Temporal (i.e., seasonal) shifts in the diet exploiting abundant prey have already been recorded for other deep‐sea elasmobranchs from that region (Preciado et al., 2009). Additionally, it can be supposed that some of the herein recorded marine fish taxa preyed by *D*. *profundorum*, including myctophids, which can harbour larval gilquiniids (Gibson et al., 2005), act as paratenic hosts.

Based on the body size and scolex morphology, some of the recorded trypanorhynch specimens were identified as immature *Aporhynchus* cf. *menezesi* (Noever et al., 2010). Previous publications recorded only one juvenile *Aporhynchus* sp. ex *D*. *profundorum* (Caira & Pickering, 2013), whereas juveniles, immature and mature adults of this genus were mainly recorded in Etmopteridae from different geographical areas (Isbert et al., 2015; Klimpel, Palm, & Seehagen, 2003; Noever et al., 2010). Therefore, it is suggested that *Aporhynchus* cf. *menezesi* cannot mature in *D*. *profundorum* and, as has been supposed for *S. spinacis*, the coexistence of *D. profundorum* and *E. spinax* in the Avilés Canyon System likely indicates an accidental infection by this cestode.

In this study, 99.6% of all larval nematodes were identified as *Anisakis* Type I (sensu Berland, 1961) (Ascaridoidea: Anisakidae). The identification and occurrence of this morphotype has been confirmed by molecular analyses in marine teleosts and sharks in previous studies from the Northeast Atlantic Ocean (e.g., Klimpel, Palm, & Seehagen, 2003; Mattiucci et al., 2004, 2008; Palm & Schröder, 2001). *Anisakis* spp. have pelagic life cycles (Klimpel et al., 2004; Klimpel & Palm, 2011), with larvae being non‐host‐specific and found in a variety of intermediate and paratenic hosts: marine invertebrates (e.g., copepods, euphausiids, cephalopods; Abollo et al., 2001; Gregori et al., 2015) and predatory vertebrates (e.g., teleosts, elasmobranchs; e.g., Busch et al., 2008; Costa et al., 2014; Klimpel, Palm, et al., 2008; Palm & Schröder, 2001), even from the deep sea. Adult stages of *Anisakis* spp. parasitize the digestive tract of pinnipeds and cetaceans (Anderson et al., 2009; Kuhn et al., 2011), and the overall infection rate in an area is influenced by the presence of its definitive hosts (Klimpel et al., 2010). The Bay of Biscay, including the Cantabrian Sea, is an area with high cetacean diversity in the Northeast Atlantic, with frequent strandings and sightings of toothed and baleen whales (de la Maza et al., 2007; Laborde Basto d'Andrade, 2008; López et al., 2004; Ruano Álvarez et al., 2007). Therefore, it is likely that the high presence of definitive hosts promotes a regular trophic transfer of this parasite, increasing infections by this nematode in all host types within the local food web, including *D. profundorum* and *E. spinax* from the same area (Isbert et al., 2015). It is assumed that high *Anisakis* infection levels observed here and in previous studies (Domingo‐Hernández et al., 2023; Isbert et al., 2015) reflect the omnipresence of *Anisakis* in hosts occurring in that area. As suggested by Isbert et al. (2015), infection levels could be further promoted by fisheries, as disposal of infected discards to opportunistic scavengers, such as *D. profundorum*, could facilitate the *Anisakis* transmission (Dimech et al., 2012; Hallett & Daley, 2011).

The processed samples of the arrowhead dogfish revealed that infracommunity mean abundance, richness and diversity were related to fish size, whereas differences in dominance (Berger‐Parker dominance index) were also explained by the factors year and sex (Table 4). Abundances of the three key discriminating parasite taxa (*Anisakis* sp., Lacistorhynchidae gen. sp. and *Deanicola* sp.) were clearly related to fish size, whereas differences in the abundance of *Deanicola* sp. could also be related to the sampling year. The latter aspect, together with the significantly higher mean abundance of Lacistorhynchidae gen. sp. in 2011, explains differing dominance and parasite infracommunity structure between the sampling years (Tables 4 and 5). In contrast, apart from the aforementioned differences in dominance between sexes, no further differences could be revealed. Several authors have indicated different behaviour and segregation of sexes in elasmobranchs (Holt et al., 2013; Sims et al., 2001; Sousa et al., 2009). In addition to the small sample size in the present study, the observed body size in both years rather indicates the presence of younger individuals, probably with less‐pronounced sex‐specific behaviour. Consequently, the absence of additional significant effects should be interpreted with caution and verified with larger and more balanced future sampling.

The observed association between the abundance of three most prevalent and abundant parasite taxa and fish size reflects parasite accumulation with host age, potentially being driven by an increased ingestion of already infected larger hosts [e.g., myctophids, squid or scavenging on moribund or dead discards in an area of frequent fishing activity (Punzón et al., 2010)]. Most of the detected *Anisakis* larvae were located in the stomach and spiral valve (86%), and the majority were encysted in the walls (94%). The cysts were often located in the transition zone between both organs and occasionally contained up to 20 individuals, indicating short feeding bouts, which has also been observed in *E. spinax* (Isbert et al., 2015). Despite this high infection by *Anisakis*, due to the benthic lifestyle of *D*. *profundorum*, it is unlikely to be a common prey for cetaceans, and the infection represents a dead end for the parasite. Although accumulation of *Grillotia* spp. larvae (Lacistorhynchidae), over host lifetime has already been observed in two elasmobranch species (Dallarés, Padrós, et al., 2017; Isbert et al., 2023; Santoro et al., 2021), data for *Deanicola* spp. are still insufficient, partly due to low host sample sizes available in previous studies (Caira & Pickering, 2013; Palm & Schröder, 2001). The present study suggests annual differences in the infection levels of adult *Deanicola* sp., which increase with the size of the host. Adult helminths in the gastrointestinal tract of their hosts are considered to be shorter‐lived than larvae (Locke et al., 2014), and their higher abundance is therefore more likely driven by the greater volume of prey consumed by larger individuals than to an accumulation over host life.

In the present study, the limited sample size, the narrow host size range and the high proportion of empty stomachs (nearly 40%) were likely insufficient to reveal potential differences between sampling years, sexes or TL for prey composition and structure. Additionally, considering that almost half of all sampled sharks can be classified as juveniles (<40 cm, Sousa et al., 2009), potential behavioural differences or spatial segregation between sexes are even more difficult to relate to the observed prey assemblage patterns. Although increasing TL showed a clear relationship with the accumulation of some parasite taxa, it could not be related to any of the detected prey items or with an increase in food consumption. It is acknowledged that size‐dependent feeding behaviour can partly explain differences in prey composition and rates of ingestion, and therefore, parasite infection patterns in fishes (e.g., Hussey et al., 2011; Timi, 2025). It can be assumed that studies with larger sample sizes and a broader range of body sizes (i.e., older individuals of *Deania* sp.) would reveal females with distinct prey composition and higher infection levels, as usually females of this genus grow larger than males (Sousa et al., 2009; Yano, 1991).

In conclusion, the arrowhead dogfish from the Avilés Canyon System appears to occupy an intermediate position within the local food web. In particular, the herein detected larval cestode parasite stages indicate its role as potential prey for larger demersal sharks in that area. The infracommunities are comparable to those of other shark species but exhibited higher diversity and richness, as previously indicated for *Deania* spp. Mean infracommunity richness, abundance, diversity and dominance appear to be related to host size, and, similarly the abundances of most prevalent and abundant taxa increased with fish length. Consequently, the accumulation of parasites during host's lifetime or/and increased ingestion rates should be considered in future studies on the biology and ecology of this host species. Regarding temporal and sexual patterns, dominance index and the abundances of *Deanicola* sp. and Lacistorhynchidae gen. sp. suggested potential interannual differences, as well as a shift in the dominance associated with sex. In addition, interannual variations in parasite infracommunity structure seems to be driven by key discriminating taxa that are largely ubiquitous in the marine environment. By contrast, the recorded diet, consisting mainly of bentho‐, bathypelagic fishes, crustaceans and cephalopods, did not reveal detectable differences between years, sexes and TL. Nonetheless, the observed patterns, particularly those suggesting interannual and intersexual differences, should be interpreted with caution, as they are highly sensitive to the restricted host size range and limited sample size of this study.

The present study provides fundamental insights into the parasite community, diet composition and their potential relationship to the arrowhead dogfish. Further studies of this kind, ideally based on larger sample sizes and including areas of the continental shelf not influenced by underwater features, are necessary to shed more light on its ecological role. Improved knowledge of parasite life cycles and their spatial occurrence would enhance their use as bioindicators, potentially revealing host trophic pathways and contributing to a deeper understanding of deep‐sea ecosystems functioning.

## AUTHOR CONTRIBUTIONS

**Wolf Isbert**: Sample processing (fish and parasites); data curation; investigation; formal analysis; writing—original draft; writing—review and editing. **Cristina Rodríguez‐Cabello**: Conceptualization; funding acquisition; project administration; design of sampling methodology and sample capture; writing—original draft; editing. **Francisco E. Montero**: conceptualization; sample processing and taxonomic identification; investigation; writing—original draft; editing; supervision. **Maite Carrassón**: Investigation; formal analysis; writing—original draft; editing. **Inmaculada Frutos**: Investigation; writing—original draft; editing. **Ana Pérez‐del‐Olmo**: Conceptualization; investigation; formal analysis; writing—original draft; writing—review and editing; supervision
